# Structural OCT Changes Following Repeated Low-Level Red-Light Therapy for Myopia Prevention

**DOI:** 10.1001/jamaophthalmol.2025.2767

**Published:** 2025-08-21

**Authors:** Zhehuan Zhang, Di Hu, Wenwen Xu, Tianchen Wu, Lu Gao, Chenhao Yang

**Affiliations:** 1 Department of Ophthalmology, Children’s Hospital of Fudan University, National Children’s Medical Center, Shanghai, China

## Abstract

This randomized clinical trial examines optical coherence tomography (OCT) changes following repeated low-level red-light therapy for myopia prevention to assess the treatment’s efficacy and safety.

Myopia represents one of the most common eye conditions worldwide, with a rapidly increasing prevalence and an increasingly earlier age at onset.^1,2^ There is a need to identify safe and clinically effective methods for delaying the onset and managing the progression of myopia. Recently, repeated low-level red-light (RLRL) therapy has gained attention as a potential treatment to control myopia.^3,4^ Our study aimed to assess the efficacy and safety of RLRL therapy for myopia control with emphasis on optical coherence tomography (OCT) changes.

## Methods

This randomized clinical trial was approved by the medical ethics committee of Children’s Hospital of Fudan University and registered in the Chinese Clinical Trial Registry (ChiCTR2400090938). The trial protocol is available in Supplement 1. Verbal consent was obtained from all participating children, and their parents provided written informed consent. The participants received comprehensive ophthalmological examinations and personalized professional consultations with ophthalmologists at no cost. Axial length, representing the optical path distance from the corneal anterior surface to the retinal pigment epithelial (RPE) layer, was measured using the IOLMaster ocular biometer (Zeiss). The study followed the Consolidated Standards of Reporting Trials (CONSORT) reporting guidelines.

A total of 86 participants aged 7 to 12 years, with refraction between −1.00 diopters (D) and +1.00 D (inclusive) in both eyes and astigmatism of less than 1.50 D, were enrolled from September 2021 to September 2023. Participants were assigned randomly to either group (1:1) using computer-generated numbers. This allocation remained masked only to the technicians and statisticians involved in the study. All intervention participants received twice-daily RLRL therapy using a 650-nm desktop device (Eyerising), administered under parental supervision. All follow-up assessments were conducted at 1, 3, and 6 months after baseline. Data analysis was performed in November 2024, using SPSS version 21.0 statistical software (IBM).

## Results

Of the 86 participants, 41 (48%) were female; the mean (SD) age was 8.43 (1.53) years. Among the 43 enrolled children in the RLRL group and 43 in the control group, the mean (SD) best-corrected visual acuities (BCVAs) measured on a logarithmic visual acuity chart (tumbling-E optotype) were 0.001 (0.008) logMAR (approximate Snellen equivalent, 20/20) and 0.004 (0.013) logMAR (approximate Snellen equivalent, 20/20), respectively. The mean (SD) axial length was 23.68 (0.64) mm in the RLRL group and 23.75 (0.64) mm in the control group. The mean (SD) spherical equivalent refraction (SER) was −0.22 (0.58) D in the RLRL group and −0.01 (0.51) D in the control group. At 6 months, 35 children (81%) in the RLRL group and 34 (79%) in the control group completed the follow-up. The axial elongation increased by 0.05 mm in the RLRL group and 0.25 mm in the control group (between-group difference, 0.20 mm; 95% CI, 0.11 to 0.28 mm; *P* < .001) (Table). The SER progression was −0.20 D in the RLRL group and −0.61 D in the control group (between-group difference, −0.40 D; 95% CI, −0.62 to −0.19 D; *P* < .001). BCVA changed by −0.001 logMAR in the RLRL group and 0 logMAR in the control group (between-group difference, 0.001 logMAR; 95% CI, −0.008 to 0.005 logMAR; *P* = .67). No severe treatment-associated adverse events occurred (Table). In the RLRL group, 3 eyes in 3 children (7%; 95% CI, 1%-15%) showed transient dome-shaped hyperreflectivity between the RPE layer and foveal ellipsoid zone on OCT (2 eyes at 1 month and 1 eye at 6 months). All 3 children had no visual symptoms, with preserved BCVA and normal fundus examination results. The hyperreflectivity resolved completely after RLRL therapy cessation (recovery time, 0.5-3 months) (Figure).

**Table.** Change From Baseline in the Repeated Low-Level Red-Light (RLRL) Therapy Group and the Control Group Over 6 Months

Measure	RLRL group	Control group	Between-group difference (95% CI)	*P* value
Change in mean at 6 mo				
BCVA, logMAR	−0.001	0	0.001 (−0.008 to 0.005)	.67
AL, mm	0.05	0.25	0.20 (0.11 to 0.28)	<.001
SER, D	−0.20	−0.61	−0.41 (−0.62 to −0.19)	<.001
SFCT, µm	30	−6.85	−36.85 (21.58 to 52.13)	<.001
Adverse events at 6 mo, No. (%)^a^	3 (7)	0	NA	.24

Abbreviations: AL, axial length; BCVA, best-corrected visual acuity; NA, not applicable; SER, spherical equivalent refraction; SFCT, subfoveal choroidal thickness.

^a^ Three eyes in 3 children in the RLRL group showed transient dome-shaped hyperreflectivity between the retinal pigment epithelial layer and foveal ellipsoid zone on optical coherence tomography (2 eyes at 1 month and 1 eye at 6 months). The hyperreflectivity resolved completely after RLRL therapy cessation (recovery time, 0.5-3 months). One case was followed up for 3 months after RLRL therapy cessation due to the COVID-19 pandemic and the associated sudden lockdown in Shanghai, China; recovery time was recorded as 3 months.

**Figure.**
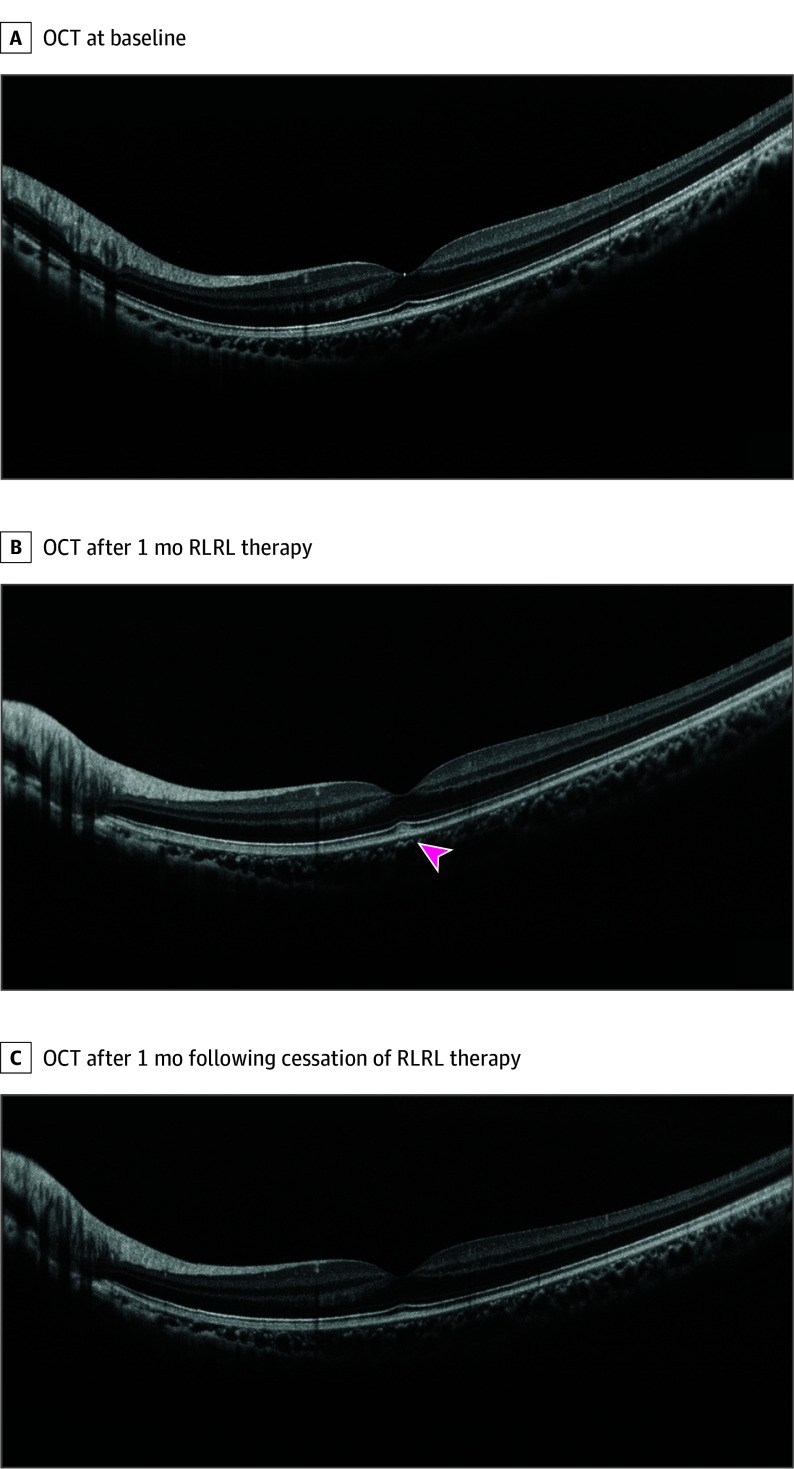
Optical Coherence Tomography (OCT) Images of the Eye With Retinal Structure Change After Repeated Low-Level Red-Light (RLRL) Therapy Over Time A, OCT image of the eye at baseline. B, OCT image of the eye with RLRL therapy after 1 month. A dome-shaped high reflection between the retinal pigment epithelial layer and the ellipsoid zone of the fovea was found (arrowhead). C, The dome-shaped reflection of the eye disappeared without RLRL therapy after 1 month.

## Discussion

This randomized clinical trial showed axial elongation and SER effects on myopia; however, 3 cases in the RLRL group showed transient foveal RPE hyperreflectivity that resolved within 0.5 to 3 months of cessation of RLRL therapy, without visual acuity decline. These findings are in contrast to the case of vision loss accompanying retinal changes reported by Liu et al.^5^ A limitation of this study is the 20% dropout rate over 6 months, which may introduce potential bias to the point estimate of our findings.

To our knowledge, this phenomenon has not been reported previously and underscores the potential importance of routine OCT scans during RLRL therapy for detection of retinal changes.

## Notes

Supplement 1. Trial Protocol

Supplement 2. Data Sharing Statement
