# Supplementary material for: Structural OCT Changes Following Repeated Low-Level Red-Light Therapy for Myopia Prevention
Source: JAMA Ophthalmol. 2025 Aug 21;143(10):876–7. doi: 10.1001/jamaophthalmol.2025.2767 (PMC12371545; doi:10.1001/jamaophthalmol.2025.2767)
Supplement: Supplement 1. — Trial Protocol [file jamaophthalmol-e252767-s001.pdf]

Structured Protocol

Repeated low-level red light for myopia prevention: A Randomized Clinical Trial

Version: V.02, 31 Argust 2021

Original Version: 20 Jun 2021

Sponsor-investigator：

Chenhao Yang, MD, PHD

Department of Ophthalmology, Children's Hospital of Fudan University, No.399 Wanyuan Road, Shanghai, 201102, China.

Phone: 086-21-64931913;

Email: ychben@hotmail.com.

TABLE OF CONTENTS

List of abbreviations

1. Trial information

1.1 Title of Project

1.2 Trial Summary

2. Background

3. Study design

3.1 Schem

3.2 Participants

3.2.1 Inclusion criteria

3.2.2 Exclusion criteria

3.2.3 Sample size

3.3 Randomization

3.4 Study intervention and termination

3.5 outcomes

3.5.1 Primary outcome

3.5.2 Second outcomes

3.6 Study procedure

3.6.1 Study visit schedule

3.6.2 Clinical examinations

4. Data Collection and Management

5. Statistical Analyses

6. Advent events

7. Ethical consideration and informed consent

7.1 Ethical consideration

7.2 Informed consent

8. Data privacy

9. Declaration of interests

10. References

List of abbreviations

AL = axial length;

BCVA = best-corrected visual acuity;

CCT = central corneal thickness;

D = diopter;

IOP = intraocular pression

UCVA = uncorrected visual acuity;

OCT = optical coherence tomography;

RCT = randomized controlled trial;

RLRL = repeated low-level red-light;

RPE = retinal pigment epithelial;

SAE = serious adverse event

SFCT = subfoveal choroidal thickness;

SER = spherical equivalent refraction;

1. Trial information

1.1 Titile of project

the efficacy and safety in children following 650 nm red-light therapy for myopia control

1.2 Trial summary

Objective: To observe the effect of low-intensity 650 nm red light on the prevention of myopia in adolescents, whether it can effectively curb myopia or slow down the development of myopia in children at high risk of myopia. we hope to provide new methods and ideas for effectively controlling myopia.

Trial Setting: The randomized controlled clinical trial is planned to be conducted between September 2021 and September 2023 at the Eye Center of the Children's hospital of Fudan University, China

Sample size: A sample size of 86 (43 in each group) is estimated after adjusting for a 20% loss to follow-up.

Inclusion criteria: 1) Children aged 7-12 years old.

2) Children with pre-myopia or mild myopia.

Exclusion criteria: 1) Participated in any myopia control clinical research trial within 3 months, used or is currently using multifocal contact lenses, atropine drugs, etc.;

2) Children with strabismus and amblyopia;

3) Congenital eye diseases, such as congenital cataracts and congenital retinal diseases;

4) Secondary myopia (such as secondary myopia caused by retinopathy of prematurity or other infant eye diseases), or myopia combined with systemic syndromes (such as Marfan syndrome);

5) People who have undergone myopia correction (such as LASIK, etc.), internal eye surgery (such as cataract extraction, intraocular lens implantation, etc.);

6) refractive media turbidity (such as keratopathy, crystal turbidity, etc.)

7) Abnormal IOP (IOP < 10 mmHg or > 21mmHg or binocular IOP asymmetry ≥5mmHg);

8) Patients with fundus retinopathy or other intraocular diseases;

9) Patients with optic nerve damage or congenital optic nerve dysfunction;

10) Have systemic diseases that may affect the eyes, such as diabetes, Down syndrome, hyperthyroidism, etc;

11) Only one eye meets the inclusion criteria;

12) Those who cannot regularly (or the guardian cannot regularly accompany the children) carry out eye examination;

13) Other reasons that the doctor considers unsuitable for inclusion in the program.

Interventions: Eligible patients will be randomly allocated with a ratio of Interventions 1:1 into 2 groups.

Follow-up plan: Each patient will undergo examinations at 1 month, 3months, 6 months, 9 months and 12 months after the enrollment.

Primary outcome: The change in axial length (AL).

Secondary outcome: The changes of uncorrected distance visual acuity (UCVA), best-corrected visual acuity (BCVA), spherical equivalent refraction (SER), subfoveal choroidal thickness (SFCT), and central cornea thickness (CCT).

2. Background

Myopia represents one of the most common eye conditions worldwide, with a rapid increasing prevalence and an earlier age of onset. It is estimated that approximately 4.758 billion people will be affected by myopia, and 938 million people will have high myopia by the year 2050.[1] Previous studies conducted on 6- to 7-year-old children have shown that the prevalence of myopia was as high as 39.5% in Guangzhou and rang from 20% to 30% in Taiwan.[2,3] The early onset of myopia has been demonstrated to increase the risk of developing high myopia.[4] To date, various clinical interventions for myopic controlling, including orthokeratology lens, multifocal soft contact lenses, and atropine, have been explored. However, the overall efficiency in controlling myopia has ranged from 30% to 60%.[5]

Recently, repeated low-level red-light (RLRL) therapy has gained significant attention as an alternative to outdoor light exposure in the treatment of myopia. A multi-center randomized controlled clinical trial of repeated low-level red-light (RLRL) irradiation for myopia control in school-age children was conducted in China from 2019 to 2020, providing evidence of the effectiveness, safety, and subject compliance of this technology in adjuvant myopic control.[6]

However, there are currently few relevant studies, and more data support is still needed, especially for children in the pre-myopia stag. Our study aims to assess the efficacy of RLRL therapy for myopia control at an early stage.

3. Study design

The study will be a 1-year, single-center, randomized, and placebo-controlled trial, with a 1:1 allocation to intervention group (RLRL treatment) or control group (observe or wear single vision glasses).

3.1 Scheme

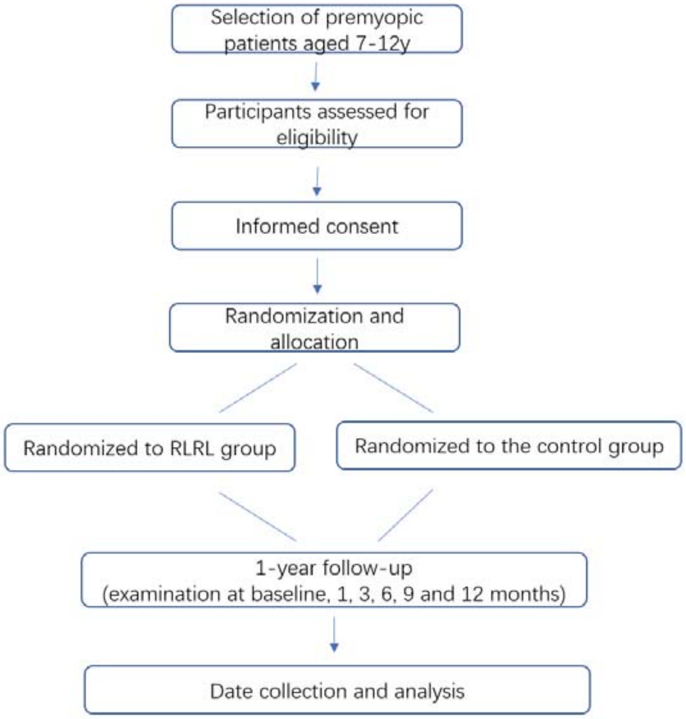

3.2 Participants

3.2.1 Inclusion criteria

1) Children aged 7-12 years old.

2) Children with pre-myopia or mild myopia.

3.2.2 Exclusion criteria

1) Participated in any myopia control clinical research trial within 3 months, used or is currently using multifocal contact lenses, atropine drugs, etc.;

2) Children with strabismus and amblyopia;

3) Congenital eye diseases, such as congenital cataracts and congenital retinal diseases;

4) Secondary myopia (such as secondary myopia caused by retinopathy of prematurity or other infant eye diseases), or myopia combined with systemic syndromes (such as Marfan syndrome);

5) People who have undergone myopia correction (such as LASIK, etc.), internal eye surgery (such as cataract extraction, intraocular lens implantation, etc.);

6) refractive media turbidity (such as keratopathy, crystal turbidity, etc.)

7) Abnormal IOP (IOP < 10 mmHg or > 21mmHg or binocular IOP asymmetry ≥5mmHg);

8) Patients with fundus retinopathy or other intraocular diseases;

9) Patients with optic nerve damage or congenital optic nerve dysfunction;

10) Have systemic diseases that may affect the eyes, such as diabetes, Down syndrome, hyperthyroidism, etc;

11) Only one eye meets the inclusion criteria;

12) Those who cannot regularly (or the guardian cannot regularly accompany the children) carry out eye examination;

13) Other reasons that the doctor considers unsuitable for inclusion in the program.

3.2.3 Sample size

The sample size estimation was estimated based on the assumption of a 2-sided α level of 0.05, 90% power. The axial elongation among Chinese children with premyopia or mild myopia was approximately 0.40 mm per year (standard deviation [SD]: 0.25 mm) according to the literature.[7-9] A 50% treatment effect was expected (reducing axial elongation by 0.20 mm). The sample size was required 34 per group. A total of at least 85 participants was obtained after adjusting for a 20% loss to follow-up. The sample size was calculated using PASS 15.0 (NCSS, Utah).

3.3 Randomization

Eligible participants will be randomized to the RLRL group or the control group. A computer-based random number table was generated with an allocation ratio of 1:1. The subjects and the clinicians were not masked to the interventions. Other investigators, and the statistician will be masked to study allocation.

3.4 Study intervention and termination

All intervention patients underwent RLRL therapy using a desktop red light therapy device (Eyerising, Suzhou Xuanjia Optoelectronics Technology, Jiangsu, China). After device dispensing, intervention patients were advised to complete treatment twice a day under their parents' supervision. Each treatment session lasted for 3 minutes, separated by at least 4 hours. In the absence of adverse effects, patients were instructed to repeat the treatment on

five days per week. Treatment dates and times were recorded via the internet, allowing researchers to monitor participant adherence.

The power of the semiconductor laser output by this device as the light source for the eyes is 1.07mW, with a wavelength of 650nm. It has been detected as a Class 1 laser by the National Quality Supervision and Inspection Center for Photoelectric Product Optical Radiation Safety, meaning that it is safe for the eyes to be directly irradiated.

Model specification of the therapeutic instrument: RS - 200 - 2A

(1) Input power ≤ 100VA, input voltage: AC10V - 240V, 50Hz/60Hz.

(2) Wavelength of the low-intensity single-wavelength red light: 650nm ± 10nm.

(3) Diameter of the low-intensity single-wavelength red light cursor: 7mm ± 3mm, and the light spot at the observation port is: 10mm ± 2mm.

(4) Light source output power: 2.0mW ± 0.5mW; at a distance of 100mm from the light source, it is 1.07 - 1.42mW.

The intervention will be terminated when the subject experiences an unexpected serious adverse reaction (including a sharp decline in vision by more than two lines or a central scotoma in the visual field), the guardian or the participant requests to withdraw from the study, or for other reasons.

3.5 Outcomes

3.5.1 Primary outcome

The primary outcome is AL change from baseline at 1 year between the two groups.

3.5.2 Second outcomes

1) the changes of UCVA and BCVA
2) the changes of SER, SFCT and CCT
3) the changes in the myopia incidence rate between the two groups.

3.6 Study procedures

3.6.1 Study visit schedule

Examination	baseline	1 months	3 months	6 months	9 months	12 months
Education and machine distribution	√	–	–	–	–	–
Informed consent	√	–	–	–	–	–
UCVA	√	√	√	√	√	√
BCVA	√	√	√	√	√	√
Noncycloplegic refraction (objective/subjective)	√	√	√	√	√	√
cycloplegic refraction (objective/subjective)	√	–	–	√	–	√
AL	√	√	√	√	√	√
Slit lamp exam	√	√	√	√	√	√
Intraocular pressure	√	√	√	√	√	√
fundus photography	√	√	√	√	√	√
optical coherence tomography (OCT)	√	√	√	√	√	√
Advent events	–	√	√	√	√	√
Machine recovery	–	–	–	–	–	√

3.6.2 Clinical examinations

1) slit-lamp examinations

   Carl Zeiss SL 115. To evaluate the eyelid, eyelashes, conjunctiva, cornea, anterior chamber, iris, pupil, lens, and anterior vitreous.

2) Axial length

   IOLMaster ocular biometer (Zeiss, Jena, Germany)

3) IOP and CCT

   NIDEK NT-530P Full Auto Tonometer (NIDEK, Tokyo, Japan).

4) Computer optometry

NIDEK APK-1 autorefractor (NIDEK, Tokyo, Japan).

5) Cycloplegic optometry

compound tropicamide eye drops were applied to both eyes four times with a 10-minutes interval. After 40 minutes, refraction data were initially measured three times and averaged using the NIDEK APK-1 autorefractor, then confirmed by an optometrist using trial lenses.

6) Fundus photography

NIDEK, AFC-330 digital fundus camera (NIDEK, Tokyo, Japan)

7) optical coherence tomography (OCT)

NIDEK RS-300 SD-OCT (NIDEK, Tokyo, Japan)

4. Data Collection and Management

During the examination, paper-based inspection forms are used to confirm the completeness of the examination. After the baseline examination is completed, the data from the paper-based inspection forms is entered into the research database. The monitor reviews each original research record form to confirm that the clinical trial data is recorded in a timely, accurate, standardized, and complete manner. Finally, the data administrator from the statistical unit checks and enters the data.

5. Statistical Analyses

For each subject, data from the right eyes were utilized for analyses. Missing data were not imputed or replaced. The changes in ocular parameters were calculated as a difference between baseline and the respective follow-up values.

Statistical analysis includes statistical descriptions and statistical inference. Continuous variables were presented as means and standard deviations, while categorical data were presented by frequency and percentage. Group differences between the RLRL and control groups were assessed using t-test or ANOVA for continuous data and the Chi-square test for categorical data. Statistical significance was set at two-sided $P<0.05$, and all analyses were performed using SPSS software version 21.0 (IBM., Chicago, IL).

6. Advent events

The intervention measure in this study is an amblyopia treatment device that has been widely used and is known to have no side effects. After using the treatment device, there may be transient reactions such as photophobia, lacrimation, mild stinging, glare, and photosensitivity. Resting with the eyes closed for a few minutes can help relieve these symptoms spontaneously. During the cycloplegic examination in the research process, some children may experience transient discomfort, such as difficulty with near reading and photophobia. These discomforts will completely disappear within 5 to 72 hours. If photophobia causes inconvenience during movement, the researchers will provide clip-on sunglasses to alleviate the discomfort. Some people may experience transient difficulty with near vision, photophobia, and other discomforts, which will typically disappear within 5 to 72 hours. For those with photophobia and blurred near vision, this study will provide sunglasses and, if necessary, reading glasses with near-addition lenses for easy reading.

Less than one in a thousand people may experience increased intraocular pressure, headache, eye pain, and vomiting (manifestations of acute angle-closure glaucoma). All subjects will be preliminarily screened with a slit lamp. Patients with a very narrow anterior chamber angle will not undergo mydriatic examination. If a subject experiences symptoms such as increased intraocular pressure, headache, or eye pain, on-site staff will promptly provide medical treatment and take referral measures.

All serious adverse events will be recorded using the SAE Case report form. Any SAE will be reported to the investigator and Institutional Ethics Committee.

7. Ethical consideration and informed consent

7.1 Ethical consideration

This trial will be submitted to the Ethics Committee of the Children's Hospital of Fudan University for review and approval. The trial protocol can only be implemented after it has been approved by the Ethics Committee, and the implementation process must comply with the regulations and requirements of relevant institutions. Researchers must submit the following information to the Ethics Committee for approval: research protocol, subject notification/informed consent form, investigator's brochure, existing safety information,

information on compensation provided to subjects, any promotional or other information related to the clinical trial distributed to subjects (including potential subjects), researcher's resume and/or professional qualifications, and any other documents that may be required. After the research protocol is approved, if any modifications are needed, they should be submitted to the Ethics Committee for re-approval before implementation.

Before the start of the study, researchers must obtain the approval or favorable opinion of the Ethics Committee/Institutional Review Board (IRB) on the written informed consent form and other written information to be provided to patients. The written approval of the Ethics Committee/IRB and the approved informed consent form and subject information must be archived together in the research documents. A written informed consent form must be obtained before implementing any specific research steps. The date when the subject participates in the study and signs the informed consent form should be recorded accordingly in the subject's medical record. Meanwhile, after the end of the trial, subjects in the control group can use the research product for free for 12 months

7.2 Informed consent

The procedures and documentation for informed consent used in the research need to be reviewed and approved by the Ethics Committee before use. The informed consent process provides participants with ongoing explanations, enabling them to make informed decisions about whether to start or continue participating in the research. Researchers will discuss the research content with the participants and their parents or guardians. They have the opportunity to ask questions before, during, and after the research. Participants and their parents or guardians have the right to be informed throughout the research process and can choose to withdraw at any time.

The Informed Consent Form for subjects provides an overview of the study, including its objectives, procedures, planned schedule, potential risks and benefits, and alternative treatments available. It also explains the rights of subjects once they participate in the study. Informed consent will be obtained from parents or guardians, and additional informed consent will be obtained from participants with age over 8 years old.

8. Data privacy

In accordance with relevant agreements, all parties involved must maintain confidentiality throughout the entire trial process. All data related to the trial may not be accessed without authorization. The private information of research subjects will be protected in reports and in the publication of any clinical research data. All records related to the identity of subjects will be kept confidential, and these materials will not be made public beyond the scope permitted by relevant laws and/or regulations. Only the subject number and the initials are recorded in the Case Report Form.

9. Declaration of interests

Any conflicts of interest from individuals involved in the design, implementation, analysis, and publication will be disclosed and managed.

10. References

1. Holden BA, Fricke TR, Wilson DA, et al. Global Prevalence of Myopia and High Myopia and Temporal Trends from 2000 through 2050. *Ophthalmology.* 2016;123(5):1036-1042.
2. He M, Xiang F, Zeng Y, et al. Effect of Time Spent Outdoors at School on the Development of Myopia Among Children in China: A Randomized Clinical Trial. *Jama.* 2015;314(11):1142-1148.
3. Lin LL, Shih YF, Hsiao CK, Chen CJ, Lee LA, Hung PT. Epidemiologic study of the prevalence and severity of myopia among schoolchildren in Taiwan in 2000. *J Formos Med Assoc.* 2001;100(10):684-691.
4. Chua SY, Sabanayagam C, Cheung YB, et al. Age of onset of myopia predicts risk of high myopia in later childhood in myopic Singapore children. *Ophthalmic Physiol Opt.* 2016;36(4):388-394.
5. Huang J, Wen D, Wang Q, et al. Efficacy Comparison of 16 Interventions for Myopia Control in Children: A Network Meta-analysis. *Ophthalmology.* 2016;123(4):697-708.
6. Jiang Y, Zhu Z, Tan X, et al. Effect of Repeated Low-Level Red-Light Therapy for Myopia Control in Children: A Multicenter Randomized Controlled Trial. Ophthalmology. 2022 May;129(5):509-519.
7. Zhu MJ, Feng HY, He XG, et al. The control effect of orthokeratology on axial length elongation in Chinese children with myopia. BMC Ophthalmol. 2014;14:141.
8. You X, Wang L, Tan H, et al. Near Work Related Behaviors Associated with Myopic Shifts among Primary School Students in the Jiading District of Shanghai: A School-Based One-Year Cohort Study. PLoS One. 2016;11(5):e0154671.
9. Ma Y, Zou H, Lin S, et al. Cohort study with 4-year follow-up of myopia and refractive parameters in primary schoolchildren in Baoshan District, Shanghai. Clin Exp Ophthalmol. 2018;46(8):861-872.
