# Supplementary material for: Structural OCT Changes Following Repeated Low-Level Red-Light Therapy for Myopia Prevention
Source: JAMA Ophthalmol. 2025 Aug 21;143(10):876–7. doi: 10.1001/jamaophthalmol.2025.2767 (PMC12371545; doi:10.1001/jamaophthalmol.2025.2767)
Supplement: Supplement 2. — Data Sharing Statement [file jamaophthalmol-e252767-s002.pdf]

Data Sharing Statement

Zhang. Structural OCT Changes Following Repeated Low-Level Red-Light Therapy for Myopia Prevention. *JAMA Ophthalmol*. Published August 21, 2025.
doi:10.1001/jamaophthalmol.2025.2767

Data
Additional Information: Chictr.org.cn (identifier, ChiCTR2400090938)
Data available: No
